# A New “Non-Traditional” Antibacterial Drug Fluorothiazinone—Clinical Research in Patients with Complicated Urinary Tract Infections

**DOI:** 10.3390/antibiotics13060476

**Published:** 2024-05-22

**Authors:** Nailya A. Zigangirova, Nadezda L. Lubenec, Vladimir B. Beloborodov, Anna B. Sheremet, Stanislava A. Nelyubina, Nataliia E. Bondareva, Konstantin A. Zakharov, Sergey I. Luyksaar, Sergey A. Zolotov, Evgenia U. Levchenko, Svetlana V. Luyksaar, Ekaterina A. Koroleva, Elena D. Fedina, Yana V. Simakova, Dmitry Yu. Pushkar, Alexander L. Gintzburg

**Affiliations:** 1 National Research Center for Epidemiology and Microbiology named after the Honorary Academician N. F. Gamaleya, 18 Gamaleya St., 123098 Moscow, Russia; 2 Medical Academy of Continuous Professional Education, 2/1 Barrikadnaya St., 125993 Moscow, Russia; 3 Accellena LLC, 88, lit.A., Sredniy pr. V.O., 199106 St. Petersburg, Russia; k.zakharov@accellena.com; 4 Department of Urology Russian University of Medicine of the Ministry of Healthcare of the Russian Federation, 4 Dolgorukovskaya St., 127006 Moscow, Russia; 5 S. P. Botkin City Clinical Hospital, Moscow Healthcare Department, 5/20 2nd Botkinsky Proezd, 125284 Moscow, Russia; 6 Department of Infectious Diseases and Virology, First Moscow State Medical University named after I. M. Sechenov, Institute of Professional Education, 18 Gamaleya St., 123098 Moscow, Russia

**Keywords:** non-traditional antibacterial agents, small molecule T3SS and flagella inhibitor, Fluorothiazinone, clinical trial, complicated urinary tract infection, antibiotic-resistant bacteria

## Abstract

In order to combat resistance, it is necessary to develop antimicrobial agents that act differently from conventional antibiotics. Fluorothiazinone, 300 mg tablet (The Gamaleya National Research Center), is an original antibacterial drug based on a new small molecule T3SS and flagellum inhibitor. A total of 357 patients with complicated urinary tract infections (UTIs) were divided into two groups and given Fluorothiazinone 1200 mg/day or a placebo for 7 days to evaluate the efficacy and safety of the drug. Additionally, all patients were given Cefepime 2000 mg/day. Fluorothiazinone with Cefepime showed superiority over placebo/Cefepime based on the assessment of the proportion of patients with an overall outcome in the form of a cure after 21 days post-therapy (primary outcome), overall outcome in cure rates, clinical cure rates, and microbiological efficacy at the end of therapy and after 21 days post-therapy (secondary outcomes). In patients who received Fluorothiazinone, the rate of infection recurrences 53 and 83 days after the end of the therapy was lower by 18.9%, compared with patients who received placebo. Fluorothiazinone demonstrated a favorable safety profile with no serious unexpected adverse events reported. The results showed superiority of the therapy with Fluorothiazinone in combination with Cefepime compared with placebo/Cefepime in patients with cUTIs.

## 1. Introduction

The development of effective antimicrobial drugs is a major challenge to overcome the looming pandemic of antimicrobial drug resistance. The 2021 annual pipeline report by the World Health Organization (WHO) describes the antibacterial clinical and preclinical pipeline as stagnant and far from meeting global needs [1]. Since 2017, only 12 antibiotics have been approved, 10 of which belong to existing classes with established mechanisms of antimicrobial resistance (AMR). Concerning that, the WHO has formulated several key innovation criteria to guide the development of effective antimicrobial medicines. These are a new target, a new class of compounds, efficacy against resistant bacteria, and reducing the rate of resistance development. According to WHO annual analyses, in 2021, there were 77 antibacterial agents in clinical development: 45 traditional direct-acting small molecules and 32 non-traditional agents. Examples of the latter are monoclonal antibodies, bacteriophages, and phage-derived enzymes; microbiome-modulating agents; immunomodulating agents; and miscellaneous agents [1]. Since antibiotics now have a limited lifespan before the drug resistance emerges, non-traditional approaches that offer new opportunities to tackle resistant bacterial infections from different angles were proposed as they can be used complementarily and synergistically or as alternatives to established therapies [2,3,4,5,6,7].

The Gamaleya Research Center of Epidemiology and Microbiology developed an original antibacterial drug with a broad spectrum of activity, effective against resistant bacteria. Fluorothiazinone (FT) is developed from a new small molecule T3SS inhibitor 2,4-disubstituted-4H-[1,3,4]-thiadiazine-5-one [8]. Fluorothiazinone works differently from antibiotics by not killing bacteria but instead inhibiting their virulence. Inhibition of virulence by Fluorothiazinone in the body leads not only to clinical improvement, but also to the eradication of the pathogen. It has previously been demonstrated that Fluorothiazinone effectively suppresses model infections caused by *Chlamydia* spp., *Pseudomonas aeruginosa*, *Acinetobacter baumannii*, *Esherichia coli*, *Klebsiella pneumoniae*, and *Salmonella enterica* in vivo, but it does not affect bacterial growth in vitro [9,10,11,12,13]. The target of the drug’s action is currently under investigation. Experimental data indicate suppression of the T3SS and the flagellum ATPase. T3SS and flagellum are essential for pathogens to implement all stages of infection, from colonization and invasion to generalization leading to sepsis and pathogen intracellular survival leading to chronic infections.

A new mechanism of action and the selected target made it possible to develop an antibacterial drug with a broad spectrum of action, due to the target versatility for a large number of pathogens [14]. Furthermore, the specific effect of the developed drug on the target only circumvents known mechanisms of antibiotic resistance and has been shown to be effective against antibiotic-resistant bacteria such as *Pseudomonas aeruginosa*, *Acinetobacter baumanii*, *Klebsiella pneumoniae*, *Escherichia coli*, *Chlamydia* spp., *Salmonella enterica*, and *Burkholderia* spp., including carbapenem-resistant bacteria that are critically prioritized by the WHO.

Pharmacokinetics studies have shown that Fluorothiazinone has high lipophilicity and low protein-binding energy, which explains the large volume of distribution and high rate of penetration into peripheral tissues and into cells. Fluorothiazinone easily penetrates into the respiratory tract, as well as genitourinary system’s organs and tissues, including the prostate, skin and soft tissues, where it accumulates in therapeutically effective concentrations [15].

Since 2017, clinical trials have been conducted with Fluorothiazinone. A favorable tolerance level of 300 to 2400 mg per day was shown in healthy volunteers, and no adverse events associated with the drug were identified.

This article presents results of the confirmation stage of clinical trail in patients with complicated urinary tract infections (UTIs) treated with Fluorothiazinone combined with Cefepime. The study was launched in 2018 (NCT03638830) and included a total of 777 patients who underwent all of the study procedures in accordance with the approved protocol. In accordance with the amendments made to the protocol, the study was carried out in three consecutive stages. During the first and second stages, a safe and effective dose was determined, and the broad-spectrum activity of Fluorothiazinone against the bacteria causing complicated urinary tract infections was demonstrated. This allowed us to conduct the conformation stage (third stage) in 2021–2023, where evaluation of the safety and efficacy of Fluorothiazinone at the most effective dose of 1200 mg per day in combination with Cefepime compared with placebo/Cefepime in patients with complicated UTIs took place.

## 2. Results

**Figure 1:**
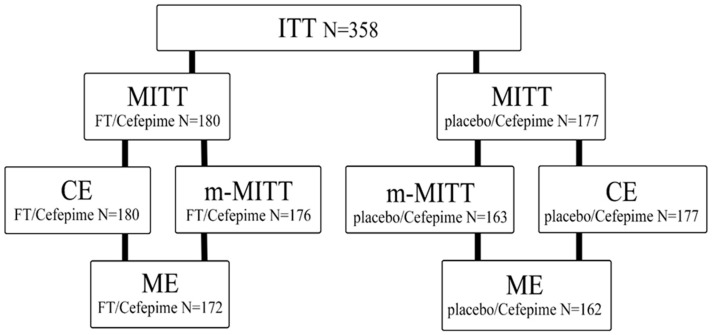
Disposition of patients enrolled in the study. ITT (intent-to-treat), MITT (modified intent-to-treat), CE (clinical evaluation), m-MITT (microbiological modified intent-to-treat), ME (microbiological evaluation).

In the period from August 2021 to January 2023, 358 patients were randomized, of which 357 received therapy with the investigational drug (Figure 1). The median duration of treatment was 7.0 days, and no deaths were observed. In the FT/Cefepime group, 1.1% (2 out of 180) of patients did not complete treatment with the study drug, compared to 1.7% (3 out of 177) in the placebo/Cefepime group.

The characteristics of randomized patients are presented in Table S1: Demographic and baseline characteristics of patients who received any dose of study drug. According to characteristics such as age, weight, height, and BMI, the groups did not significantly differ from each other.

All patients included in the study had suspected or documented complicated UTIs. All subjects also met other inclusion criteria and did not have non-inclusion criteria. Infection with Gram-negative pathogens took place in 84.6% (291 out of 344) of patients. The most common Gram-negative pathogens at the baseline level were representatives of the *Enterobacterales* family and amounted to 74.2%. Among them, *Escherichia coli* dominated (52.9%; 182/344), and *Klebsiella pneumoniae* had the second highest prevalence (26.5%; 91/344), followed by *Proteus* spp. (6.7%; 23/344). Other Gram–negative pathogens were isolated in a smaller percentage—7.6% (26/344); the detectability of *Pseudomonas aeruginosa* was 6.1% (21/344). The most common Gram-positive pathogens were *Enterococcus* spp. (22.4%; 84/344) and *Staphylococcus* spp. (1.7%; 6/344).

### 2.1. Primary Outcome

The analysis of the primary efficacy endpoint included 357 patients, 180 in the FT/Cefepime group and 177 in the placebo/Cefepime group. The primary outcome was the difference in the proportion of patients with a general response in the form of a cure after 21 days post-therapy (the TOC visit) in the group of patients meeting the MITT (modified intent-to-treat) criterion. The choice of such an indicator includes a wider population and reflects an approach to evaluating therapy in which it is the clinical efficacy of the treatment that comes to the fore, regardless of whether the pathogen has been isolated or not. Indeed, the clinical outcome is obviously more important for the patient than the results of diagnostic procedures and tests.

The primary outcome was evaluated based on the complete elimination of the initial signs and symptoms present during screening (clinical cure) and a positive microbiological response according to the protocol at the TOC visit.

In patients of the MITT population, an overall positive outcome in the form of a cure was observed in 136/180 (75.6% (95% CI (confidence interval): 68.6–81.6)) patients in the FT/Cefepime group and in 90/177 (50.8%; (95% CI: 43.0–58.1)) patients in the placebo/Cefepime group. Therefore, the frequency of achieving overall outcome in the form of a cure on the 21st days after the end of therapy in the FT/Cefepime group was 24.8% higher than in the placebo/Cefepime group (Table 1). Data analysis showed that the lower limit of the one-sided 97.5% confidence interval for the difference between values of 75.6% and 50.8% was 14.7%, which exceeded the value of clinically significant differences of 0%. The presented results allowed us to accept an alternative hypothesis (H1): FT/Cefepime has superiority in comparison with placebo/Cefepime in the treatment of patients with complicated urinary tract infections.

### 2.2. Secondary Outcomes

Similar results on the overall response were obtained for the secondary outcome, specifically the proportion of patients with an overall outcome in the form of a cure at the TOC visit in the group of patients meeting the m-MITT (microbiological modified intent-to-treat) criterion, i.e., those who had an isolated pathogen (including *P. aeruginosa*, *E. coli*, and *Enterococcus* spp.) in urine at baseline. The difference between the groups was 21.5% (*p* = 0.0001), which showed the superiority of therapy in the group of patients receiving FT/Cefepime (Table 2).

The proportions of patients with a clinical cure response were compared in the population of patients meeting the MITT criterion; in the group of patients meeting the m-MITT criterion; in the population of patients suitable for clinical evaluation, CE; in the population of patients suitable for microbiological validation, ME (microbiological evaluation); and at the TOC visit. The proportion of patients in all four analyzed populations with a clinical cure response at the 21st day after the end of treatment was significantly higher in patients who received FT/Cefepime compared with those who received placebo/Cefepime (Table 2). Analysis showed that the confidence intervals did not overlap, and the clinical cure proportion was statistically greater (*p* < 0.0001) in the FT/Cefepime group compared to the same response in the placebo/Cefepime group, which may indicate greater efficacy of Fluorothiazinone at a dose of 1200 mg/day, in combination with Cefepime.

The microbiological efficacy was evaluated for the m-MITT and ME population in accordance with the criteria for evaluating the microbiological response at the TOC visit in accordance with the protocol. The positive microbiological response consisted in the eradication of the baseline pathogen and/or the presumed stable microbiological eradication. Microbiological recurrence and/or colonization were assessed as microbiological inefficacy. In both studied populations, microbiological efficacy was significantly higher in the FT/Cefepime group compared to the placebo/Cefepime group, by 13.5% for the m-MITT population and by 14.9% for the ME population (Table 2).

During follow-up on day 53 and day 83 after the end of treatment, clinical efficacy and recurrence frequency were evaluated. In the population of patients suitable for clinical evaluation who received FT/Cefepime, a significantly lower number (by 14.9%) of infection recurrences was revealed, i.e., (2/179 (1.1% (CI: 0.1–4.0)) compared with placebo/Cefepime, i.e., (28/175 (16.0% (CI: 10.9–22.3)) (*p* < 0.0001) at 53 days after the end of therapy. The same indicator on the 83rd day of the follow-up also remained significantly lower, by 6.3% in patients receiving FT/Cefepime (3/178 (1.7% (CI: 0.3–4.8%)) compared with placebo/Cefepime (14/175 (8.0% (CI: 4.4–13.1)) (*p* < 0.012). At the same time, the total number of recurrences recorded at the LFU visits was significantly lower, by 18.9% in patients receiving FT/Cefepime (5/179 (2.8% (CI: 0.9–6.4%)) compared with placebo/Cefepime (38/175 (21.7% (CI: 15.8–28.6)) (*p* < 0.0001).

### 2.3. Additional Outcomes

The analysis of the correlation between the microbiological response and the clinical response showed that microbiological recurrence or colonization often led to clinical inefficacy or clinical recurrence at the TOC visit (Table 3).

The therapy efficacy was also evaluated immediately after the end of treatment at the EOT visit. It was noted that Cefepime, which patients received at a dose of 2000 mg/day IM or IV, was highly effective in the treatment of the acute course of complicated UTIs, leading to effective eradication of pathogens and clinical cure. However, even against the background of such an effective action of Cefepime at the EOT visit, the best values of microbiological and clinical responses were observed in the m-MITT population in the group of patients receiving Fluorothiazinone additionally. Therefore, the persistence of the baseline pathogen (>10^3^ CFU/mL) in the placebo/Cefepime group was 14.1% (23/163), which was almost two times higher than in the FT/Cefepime group, being 8.5% (15/176). Analysis of the clinical response in the CE (clinical evaluation) population at the therapy completion visit (EOT visit) in the group of patients receiving FT/Cefepime showed complete absence of cases of treatment inefficacy and 3.3% (6/180) of cases with an uncertain result due to insufficient data to determine cure or inefficacy in the patient. At the same time, in the group of patients receiving placebo/Cefepime, 5.6% (10/177) of cases of treatment inefficacy and 7.3% (13/177) of cases with an uncertain result were observed. Pathogen-specific eradication assessments showed that at the EOT and the TOC visits, microbiological efficacy was higher in the FT/Cefepime group (Table 4).

### 2.4. Safety Report

Safety analysis was conducted in population of patients who received any amount of the investigational drug. Among 358 people who took part in the third stage of study, in total, 63 adverse events (AE) related to the somatic status and changes in the laboratory parameters of patients were registered during the study. No serious AE or deaths were observed. When comparing the rate of patient withdrawal from the study due to insufficient efficacy of the therapy, no significant differences were found between the groups; in both groups, one patient dropped out for this reason.

The number and percentage of AE by severity, by cause-and-effect relationship with the drug, by actions taken when AE occurred, and by outcome, as well as by systemic organ classes (SOC) by MedDRA, are shown in Table 5. In patients of the FT/Cefepime group, 37 out of 63 AE (58.7%) and in patients of placebo/Cefepime group, 26 out of 63, were registered (41.3%). The incidence of AE per patient in the FT/Cefepime group was 0.204, in the placebo/Cefepime group—0.147. For all systems, the AE in the analyzed groups did not significantly differ from each other (Table 5).

## 3. Discussion

### 3.1. Limitations

The clinical study had a number of limitations. Thus, the study included a population of patients with one nosology—complicated urinary tract infections. This nosology is one of the most important infectious and inflammatory diseases caused by *Pseudomonas aeruginosa*, *Escherichia coli*, *Klebsiella pneumoniae*, and *Enterococcus* spp., including those resistant to antibacterial drugs. Data obtained during the clinical study can be extrapolated to other nosologies caused by these pathogens, as well as to others for which preclinical studies have shown efficacy and distribution of Fluorothiazinone to most organs and tissues [9,12,13].

In such trials, a control group is usually a drug approved for use in a studied condition. This design could not be applied in this study due to a unique mechanism of action of Fluorothiazinone. Fluorothiazinone has been evaluated in combination with Cefepime, but combination with other antibiotics has not been studied. Due to the mechanism of action of Fluorothiazinone and according to the results of preclinical studies, its pharmacodynamic or pharmacokinetic interactions are not expected, and low potential for drug–drug interactions (DDIs) has been shown.

The use of Fluorothiazinone as a monotherapy has not yet been assessed, which can be considered a further step of the clinical development of Fluorothiazinone, 300 mg tablets (Gamaleya Research Center of Epidemiology and Microbiology, Ministry of Health of the Russian Federation).

### 3.2. Interpretation

The drug Fluorothiazinone is a small molecule inhibitor of key virulence factors of a wide range of pathogenic bacteria—type III secretion system (T3SS) and flagellum. According to the mechanism of action, Fluorothiazinone belongs to the category of “non-traditional antibacterials” as defined by the WHO.

According to the 2021 annual pipeline report by the WHO, overall, 32 non-traditional antibacterials are under active clinical development: 6 antibodies, 9 bacteriophages and phage-derived enzymes, 10 microbiome-modulating agents, 1 immunomodulating agent, and 6 agents in the miscellaneous category. Most non-traditional drugs are being tested and are intended for use in combination with standard antibiotics.

From a clinical point of view, combination therapy regimens are used to treat complicated infections due to the fact that no single antibiotic in the empirical regimen is capable of suppressing the entire spectrum of possible pathogens. Combination regimens typically use antibacterial drugs with different mechanisms of action. Therefore, the addition of Fluorothiazinone, which has a unique mechanism of action aimed at suppressing key virulence factors—T3SS proteins and flagellum, which is different from other known antibacterial drugs, can have a cumulative effect in combination with them, increasing microbiological and clinical efficacy, which has been proved in this study. The suppression of the virulence of uropathogens will allow for more effective treatment of severe complicated UTIs, as well as reduce the number of antibacterial drugs used. It is well known that drugs under development with an analogous mechanism of action are intended for use as part of complex therapy, also in the case of evidence of effectiveness, as monotherapy and as prophylaxis [16,17].

In the conformation phase of our clinical trial, the efficacy of Fluorothiazinone was studied in patients with complicated UTIs during an exacerbation of the disease that required hospitalization. In this regard, all patients included in the study received the same standard antibacterial therapy—Cefepime 2000 mg per day. The study of the pharmacokinetic interaction of Fluorothiazinone and Cefepime conducted in preclinical studies did not reveal statistically significant changes in the pharmacokinetic parameters of both drugs. Also, in vitro and in vivo experiments showed the absence of a pharmacodynamic interaction of Fluorothiazinone with Cefepime and with antibiotics of different classes, since Fluorothiazinone and Cefepime act by different mechanisms [18,19].

In this study, the value of the primary outcome was defined as the proportion of patients with an overall cure outcome at the TOC visit in the group of patients meeting the MITT criterion. The value of the mentioned outcome in the FT/Cefepime group was 75.6% (97.5% CI 68.6–81.6%) and in the placebo/Cefepime group—50.8% (97.5% CI 43.0–58.1%). Therefore, the positive outcome value was found to be clinically significant.

Disease incidence and recurrence are the main indicators of the low efficacy of the antibacterial therapy in the treatment of chronic infections. Achieving effective eradication of the pathogen may be difficult due to at least two factors: its antibiotic resistance and intracellular localization.

Uropathogenic *E. coli* (UPEC) has been reported to replicate in the epithelial cells of the bladder in mice [20]. This suggests that bladder epithelial cell invasion is the crucial step for UPEC proliferation. By infiltrating the bladder epithelial cells, UPEC forms a biofilm-like intracellular bacterial community [21]. This mechanism enables UPEC to avoid host defense and antibiotic therapy and allows for recurrent or chronic infections. Intracellular colonization by UPEC has also been demonstrated in the epithelial cells of the prostate. A study has shown that UPEC strains are able to attach to and infiltrate the normal human prostate cells with high efficiency [22].

Fluorothiazinone showed high efficacy in reducing clinical recurrence of cUTIs 2 and/or 3 months after the start of therapy. The detected efficacy against recurrences was likely due to the successful eradication of the pathogens during Fluorothiazinone therapy. The microbiological eradication is well explained by the properties of the drug. Firstly, Fluorothiazinone has proven its antibacterial efficacy against multidrug-resistant pathogens during the development of the drug. Secondly, Fluorothiazinone easily penetrates membranes and distributes intracellularly due to its lipophilic properties. This has been demonstrated in the proliferation suppression of the obligate intracellular pathogens, *C. trachomatis*, and in the intracellular development of *E. coli* [8,12,20].

The clinical importance of Fluorothiazinone lies in its ability to increase the efficacy of the treatment of infections caused by antibiotic-resistant pathogens, reducing the number of recurrences of chronic infections that are practically untreatable with antibiotics.

Fluorothiazinone is a drug with a favorable safety profile. In clinical studies on healthy volunteers, it has been shown that Fluorothiazinone has a favorable level of tolerability with a one-day administration at a dose of 300 to 2400 mg/day and with a course intake (7 days) at doses of 1200 and 1800 mg/day. In preclinical studies, it was shown that Fluorothiazinone belongs to low-toxic drugs, and LD_50_ was more than 5 g/kg. The study of chronic toxicity in rats and rabbits indicated the safety of Fluorothiazinone. In the conducted study, the observed adverse events were predominantly of mild severity; no serious adverse or unexpected events were reported. Important identified risks of headache, sinus tachycardia, and sinus bradycardia were identified in a randomized clinical trial. These risks are expected, unpreventable, and do not have a direct impact on public health.

A significant problem of antibiotic therapy is the negative effect on the normal human microflora [23,24,25]. The specificity of action of antivirulent drugs towards the particular virulence factor ensures the absence of side effects and damaging effects on microbiome. For Fluorothiazinone, it was shown in animals and healthy volunteers that during the course of therapy, there was no disturbance in the composition of the intestinal microflora.

The developed drug Fluorothiazinone meets the WHO criteria for innovation in the development of effective antibacterial drugs. The mechanism of action of Fluorothiazinone differs from antibiotics because it does not kill bacteria but inhibits their virulence [26,27,28]. This mechanism of action fundamentally reduces the risk of developing resistance due to the absence of strict selective pressure. The drug was developed based on a new class of chemical compounds for which resistance mechanisms are not known. Fluorothiazinone is effective against multidrug-resistant bacteria, including those of critical priority specified by the WHO list. The global benefit of treating infectious diseases with virulence factor inhibitors is reducing the spread of the pathogen strains resistant to antibacterial drugs, which is consistent with the WHO Global Target [29].

## 4. Materials and Methods

### 4.1. Trial Design

A multicenter, randomized, blind, placebo-controlled study in parallel groups was conducted evaluating the safety and efficacy of Fluorothiazinone in combination with Cefepime compared with placebo in combination with Cefepime in the treatment of patients with cUTIs. The study was conducted in 14 trial sites on the territory of the Russian Federation from 2018 to 2023 (Table S2: List of the trial sites and the principal investigators). The trial was carried out in accordance with the permission to conduct the study no. 389 dated 3 August 2018 (Ministry of Health of the Russian Federation) and amendments “№4098616-20-1/ПП” from 11 April 2019, “№4127433-20-1/ПП” from 27 February 2020, “№4134730-20-1/ПП” from 13 May 2020, “№4181704-20-1/ПП” from 20 August 2021, “№4215552-25-2/ПП” from 23 June 2022, and “№4229674-25-2/ПП” from 3 November 2022. The study protocol was reviewed and approved by the Ethics Committee of the Ministry of Health of the Russian Federation and the ethics committee of each trial site, and written informed consent was obtained from each patient before inclusion in the study.

**Figure 2:**
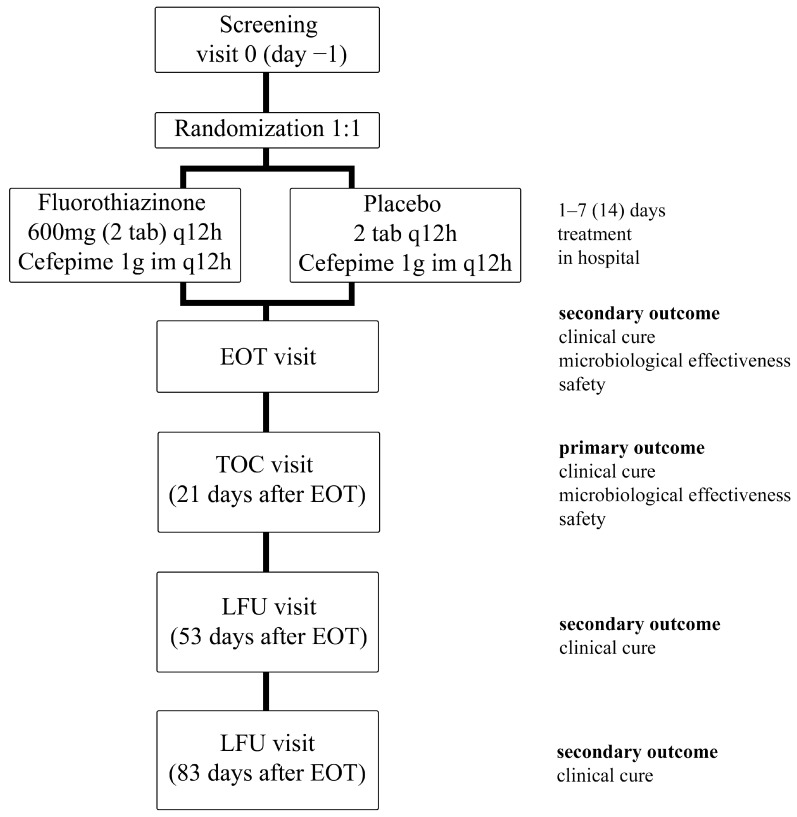
Study design. EOT (end-of-therapy) visit, TOC (test-of-cure) visit, LFU (late-follow-up) visit.

The patients were divided into 2 groups and received Fluorothiazinone at a dose of 1200 mg/day or placebo along with Cefepime at a dose of 2000 mg/day. The duration of the therapy period was 7 days, and according to the indications, i.e., in the absence of therapeutic efficacy, up to 14 days. All patients were observed at the end-of-therapy (EOT) visit and in the follow-up period, up to 90 days after the start of the study. Therapy efficacy (clinical and microbiological) was evaluated at the test-of-cure (TOC) visit (the 21st day post-therapy) and clinical recurrence at the late follow-up (LFU) visits (53 and 83 days after the last dose of the study drug) (Figure 2).

### 4.2. Participants

The study included patients aged ≥18 years old with the diagnosis of complicated urinary tract infection requiring hospitalization and parenteral antibacterial therapy, if patients had at least two of the following symptoms within 24 h before screening: fever > 38 °C, chills; nausea or vomiting; dysuria, frequent urination or imperative urge to urination; pain in the lower abdomen; acute flank pain (appeared within 7 days before randomization); soreness in the rib-vertebral angle; leukocyturia: >10 cells in the field of view during microscopy of urine sediment, a positive reaction to leukocyte esterase in urine (in the absence of leukocytes in the sediment). The inclusion criteria were also cUTIs in the case of structural or functional abnormalities of the urinary tract and urinary tract obstruction; instrumental interventions or surgeries on the urinary tract; recurrent cUTIs; and high risk of cUTIs caused by resistant strains of *P. aeruginosa* and *E. coli*, as well as the ability to understand the requirements for study participants, to give written consent to participate in the study, and to follow the procedures specified by the Study Protocol.

### 4.3. Interventions

Patients were randomly assigned to one of two groups in a 1:1 ratio to receive either Fluorothiazinone at a dose of 1200 mg/day or placebo. All patients, regardless of the group, also received Cefepime, a powder for preparing a solution for intravenous and intramuscular administration of 1000 mg. Cefepime was dissolved in 2.4 mL of water for injection or 0.9% sodium chloride solution or 0.5–1% lidocaine hydrochloride solution and administered intramuscularly into the upper outer quadrant every 12 h for 7 days. Fluorothiazinone or placebo were administered at about the same time with Cefepime every day, twice a day, 2 tablets in the morning and evening. It was allowed for them to take Fluorothiazinone or placebo within 30 min after the injection of Cefepime. The duration of therapy was 7 days. In the case of indications for the extension of antibacterial therapy, the duration of treatment could have been increased to 14 days.

### 4.4. Outcomes

#### 4.4.1. Study Populations

Evaluation of both efficacy and safety depended on the populations of study. In our research, we have provided the following populations of study:

- MITT—patients who met the ITT (intent-to-treat) criterion and received any amount of the drug specified by the study protocol;
- m-MITT—included patients who met the MITT criteria and who had any pathogenic microorganism detected in their urine, including *P. aeruginosa*, *E. coli*, and *Enterococcus* spp., at baseline;
- The population of patients eligible for CE included patients who met the MITT criteria and met the criteria for suitability evaluation (met the basic inclusion criteria, had no non-inclusion criteria, received ≥80% of the estimated doses, and lacked any other factors that could interfere with the efficacy assessment);
- The population of patients suitable for ME included patients who met the m-MITT criteria and CE criteria and who had a properly collected urine sample for culture and a suitable urine culture result for evaluation at the EOT or the TOC visits.

#### 4.4.2. Descriptions of Responses

Based on the assessment of signs and symptoms, the clinical research physician (CRP) selected one of the following clinical responses at the TOC visit:

- *clinical cure*—compliance with the criteria of clinical cure and absence of signs of cUTIs at visit;
- *recurrence*—clinical cure after completion of therapy at the EOT visit, but appearance of new signs and symptoms of complicated UTIs at the TOC visit, causing the patient to be in need for antibiotic therapy for complicated UTIs;
- *clinical inefficacy*—the symptoms of the complicated UTI that were present at the time of inclusion in the study were not completely resolved at the EOT and TOC visits, or new symptoms developed and antibiotic therapy beyond the scope of the study is required, or death occurred;
- *clinically uncertain response*—there are insufficient data to determine cure or inefficacy to the patient.

The microbiological response in each patient was determined based on the results of blood and urine cultures as one of the following outcomes at the TOC visit:

- *microbiological eradication*—microbiological eradication;
- *presumed sustained microbiological eradication*—urine culture at the TOC visit was not performed or was lost, but the patient meets the clinical criteria for clinical cure;
- *microbiological recurrence*—urine culture performed at the TOC visit showed ≥104 CFU/mL of any of pathogenic bacteria identified at baseline;
- *microbiologically uncertain response*—there was no urine culture at the TOC visit, or urine culture was unable to be interpreted for any reason at the follow-up visit, or urine culture was recognized as contaminated.

Additional microbiological response included the following:

- *colonization*—isolation of a new pathogenic bacteria at a concentration of ≥10^5^ CFU/mL (different from pathogenic microorganisms found at baseline) from urine culture of the patients who met the criteria of clinical cure.

The overall outcome was determined based on the clinical response and microbiological response as one of the following: positive outcome, no effect, and uncertain outcome (Table S3: Evaluation criteria for the overall outcome at the TOC visit). We used all of the definitions of treatment response to assess the achievement of defined study endpoints.

#### 4.4.3. Descriptions of Outcomes

The primary outcome was the proportion of patients who achieved an overall outcome of complete resolution of the baseline signs and symptoms present at screening (clinical cure) and eradication of pathogens in urine (microbiological response) at the test-of-cure (TOC) visit (after 21 days post-therapy) in the patients of the MITT population. All patients corresponding to this population had documented complicated UTIs according to clinical and/or microbiological criteria for exacerbation of complicated UTIs.

Secondary efficacy endpoints included the following:

- proportion of patients with an overall outcome in the form of a cure at TOC visit in the group of patients meeting the m-MITT criterion, i.e., having isolated pathogen (including *P. aeruginosa*, *E. coli*, and *Enterococcus* spp.) in urine at baseline;
- proportion of patients with a clinical cure response at TOC visit in the population of patients treated with drugs specified by the study protocol, i.e., meeting the MITT criterion; in the group of patients with an isolated pathogen (including *P. aeruginosa*, *E. coli*, and *Enterococcus* spp.) in urine at baseline, i.e., corresponding to the m-MITT criterion; populations of patients suitable for clinical evaluation (CE); populations of patients suitable for microbiological evaluation (ME);
- proportion of patients with a clinical response in the form of recurrence based on combined data from LFU visits in the patient population suitable for clinical evaluation (CE);
- proportion of patients with a response in the form of microbiological eradication for all isolated pathogens in the MITT, m-MITT, and ME populations at the EOT visit and at the TOC visit;
- proportion of patients with a microbiological response in the form of recurrences and colonization, represented by groups, in the ME population at the TOC visit;
- frequency of withdrawal from the study due to insufficient efficacy of therapy.

### 4.5. Sample Size Calculation

Calculation of the sample size according to the formula given in the work of Chow S. et al. [30,31] provided the number of patients in each of the two groups, which considering the uniform distribution, was equal to 153. Values for calculating the sample size: the magnitude of the difference between frequencies ε = 12%, the margin of superiority is 0%, α (type 1 error) 0.05, β (type 2 error) 0.2. Taking into account the screening errors, the incidence of infection caused by *P. aeruginosa*, *E. coli*, and *Enterococcus* spp., as well as dropouts during the study, it was necessary to include at least 351 patients to randomize about 332 patients.

The design of the study was aimed at demonstrating the superiority of Fluorothiazinone compared with placebo in combination with Cefepime (standard antibacterial therapy).

### 4.6. Randomization

In order to minimize bias, randomization into treatment groups was carried out without stratification by the clinical center [32]. The subjects were randomized on the day of administration of the investigational drug or placebo.

The randomization scheme was prepared prior to the start of the study using validated software. The inclusion of patients into the study and the prescription of the treatment in accordance with the randomization scheme was carried out by the clinical research physician (CRP).

To minimize bias, the clinical study of the efficacy and safety of Fluorothiazinone was conducted using placebo, which did not differ in appearance, shape, nor color and completely imitated the study drug. Fluorothiazinone and placebo were taken according to the same scheme.

The study by type was single-blind, i.e., it was blinded only to the patient; the researcher was aware of which drug the patient was taking.

### 4.7. Statistical Analyses

To determine the type of distribution (normality) of quantitative data, the Shapiro–Wilk test was used (with a sample size of up to 50 people) or the Kolmogorov–Smirnov test (with a sample size of 50 or more people). Ordinal, categorical, and qualitative data are represented in the form of absolute frequencies, relative frequencies, and 95% confidence interval (CI) of a proportion (lower limit (LL) of CI,% and upper limit of (UL) CI,%) calculated using the Clopper–Pearson exact method.

For the primary endpoint, the comparison of patient groups was performed by calculating a one-sided 97.5% CI for the overall cure rate difference. The CI was calculated using the statistical Z-test with the Yates correction for continuity. If group comparison indicated that the lower limit of 97.5% CI for the difference in the m-MITT population was greater than 0%, it was concluded that Fluorothiazinone at the appropriate dose in combination with Cefepime was superior to the placebo in combination with Cefepime.

For each secondary endpoint, the absolute and relative frequencies were presented along with the two-sided 95% CI according to Clopper–Pearson [33].

The efficacy of therapy in the study groups was determined using Fisher’s exact p test or Pearson’s chi-squared test (χ^2^ test) with Yates’ correction, depending on the number of expected frequencies and by comparing confidence intervals (without calculating the difference of proportions).

If the values (of Fisher’s exact test or Pearson’s chi-squared test) were significant (*p* ≤ 0.05) or the confidence intervals of the parts for the groups did not overlap (the lower limit of the CI of group 1 was greater than the upper limit of group 2), it was decided that Fluorothiazinone in combination with Cefepime was more effective than the placebo in combination with Cefepime. The applied significance level was *p* ≤ 0.05. The replacement of missing data with model estimates was not provided.

The 0% margin of superiority was used based on retrospective data on the therapeutic effect of antibiotics. Such margin is stable and can sufficiently confirm the clinically significant therapeutic effect of Fluorothiazinone in combination with standard antibacterial therapy in the treatment of complicated UTIs.

## 5. Conclusions

Fluorothiazinone demonstrated a favorable safety profile, with no serious unexpected adverse events reported. The study showed the superiority of Fluorothiazinone/Cefepime compared with the placebo/Cefepime supported by the analysis of primary and secondary outcomes, which were clinical cure, microbiological efficacy, and recurrence rates at the TOC and the LFU visits in patients with complicated urinary tract infections.

## Figures and Tables

**Table 1:** Primary outcome.

Group	N, Patients	Absolute Frequency with Cure, Patients	Means, %		
			Relative Frequency	LL (Lower Limit) 95% of CI	UL (Upper Limit) 95% of CI
FT/Cefepime	180	136	75.6	68.6	81.6
Placebo/Cefepime	177	90	50.8	43.0	58.1

**Table 2:** Clinical cure and microbiological response.

Response/Population	n/N (% (CI)) FT/Cefepime	n/N (% (CI)) Placebo/Cefepime	*p* ^1^
**Day 21 after the last antibiotic dose**			
Overall success/m-MITT	135/176 (76.7 (95% CI: 69.7–82.7))	90/163 (55.2 (95% CI: 47.2–63.0))	0.0001 *
Clinical cure/MITT	159/180 (89.2 (95% CI: 83.7–93.4))	110/163 (67.5 (95% CI: 59.7–74.6))	0.0001 *
Clinical cure/m-MITT	157/176 (89.2 (95% CI: 83.7–93.4))	110/163 (67.5 (95% CI: 59.7–73.6))	0.0001 *
Clinical cure/CE (clinical evaluation)	159/180 (88.3 (95% CI: 82.7–92.6))	123/177 (69.5 (95% CI: 62.1–76.2))	0.0001 *
Clinical cure/ME (microbiological evaluation)	153/172 (89.0 (95% CI: 83.3–93.2))	109/162 (67.3 (95% CI: 59.5–74.4))	0.0001 *
Microbiological efficiency/m-MITT	134/176 (76.1 (95% CI: 69.1–82.2%))	102/163 (62.6 (95% CI: 54.7–70.0%))	0.009 *
Microbiological efficiency/ME	134/172 (77.9 (95% CI: 71.0–83.9%))	102/162 (63.0 (95% CI: 55.3–70.6))	0.004 *
Recurrence/MITT	9/180 (5 (95% CI: 2.3–9.3%))	29/177 (16.4 (95% CI: 11.3–22.7%))	0.001 *
**Day 53 after the last antibiotic dose**			
Recurrence/CE	2/179 (1.1 (95% CI: 0.1–4.0))	28/175 (16.0 (95% CI: 10.9–22.3))	<0.0001 *
**Day 83 after the last antibiotic dose**			
Recurrence/CE	3/178 (1.7 (95% CI: 0.3–4.8))	14/175 (8.0 (95% CI: 4.4–13.1))	0.012 *
**Day 53+ 83 after the last antibiotic dose**			
Recurrence/CE	5/179 (2.8 (95% CI: 0.9–6.4))	38/175 (21.7 (95% CI: 15.8–28.6))	<0.0001 *

^1^—*p*-value in χ^2^ test; *—differences assessed using χ^2^ test were statistically significant (*p* ≤ 0.05).

**Table 3:** Square matrix of microbiological and clinical response at the EOT and the TOC visits.

Response	Clinical Cure	Clinically Uncertain Outcome	Clinical Inefficacy
	The EOT visit		
Microbiological eradication	159	4	0
Colonization	16	2	0
Microbiological persistence	14	2	0
	The TOC visit		
Microbiological eradication	146	3	2
Colonization	12	0	7
Microbiological recurrence	12	3	12

**Table 4:** Eradication of the most common pathogens of infection at the EOT visit and on the 21st day after the end of treatment (the TOC visit).

Pathogens	Eradication at the EOT Visit		
	n/N (%) FT/Cefepime	n/N (%) Placebo/Cefepime	Treatment Difference, %
*Enterobacter*	11/13–84.6	9/11–81.8	2.8
*Enterococcus faecalis*	58/70–82.9	35/50–70	12.9
*Escherichia coli*	105/109–96.3	79/93–84.9	11.4
*Klebsiella pneumoniae*	47/49–95.9	32/39–82.1	13.8
*Proteus* spp.	12/14–85.7	12/16–75.0	10.7
*Pseudomonas* spp.	11/12–91.7	11/16–68.8	22.9
*Staphylococcus*	24/29–82.8	22/32–68.8	14
*Streptococcus*	15/18–83.3	10/17–58.8	24.5
	Eradication at the TOC visit		
*Acinetobacter* spp.	3/4–75.0	4/8–50	25
*Enterobacter* spp.	11/12–91.7	7/11–63.6	28.1
*Enterococcus faecalis*	54/70–77.1	33/61–54.1	23
*Escherichia coli*	94/109–86.2	70/93–75.3	10.9
*Klebsiella pneumoniae*	42/49–85.7	27/39–69.2	16.5
*Proteus* spp.	13/14–92.9	14/16–87.5	5.4
*Pseudomonas* spp.	10/12–83.3	12/16–75	8.3
*Staphylococcus* spp.	25/29–86.2	23/32–71.9	14.3
*Streptococcus* spp.	17/18–94.4	14/17–82.4	12

**Table 5:** Patients with any treatment-emergent adverse event.

SOC MedDRA	FT/Cefepime, N = 180 n (%)	Placebo/Cefepime, N = 177 n (%)
Patients with any treatment-emergent adverse event	37 (58.7)	27 (41.3)
Headache	6 (3.3)	5 (2.8)
Blood creatinine increased	6(3.3)	1 (0.6)
Blood urea increased	4 (2.2)	2 (1.1)
Cough	1 (0.6)	3 (1.7)
Nasal congestion or rhinorrhea	2 (1.1)	1 (0.6)
Diarrhea	2 (1.1)	1 (0.6)
Kidney and urinary disorders	2 (1.1)	1 (0.6)
Creatine phosphokinase increased	1 (0.6)	2 (1.1)
Skin and subcutaneous tissue disorders	1 (0.6)	2 (1.1)
Erythrocyte sedimentation rate increased	2 (1.1)	0
General disorders and administration site conditions	2 (1.1)	0
Abdominal pain	1 (0.6)	1 (0.6)
Aspartate aminotransferase increased	1 (0.6)	1 (0.6)
Blood bilirubin increased	1 (0.6)	1 (0.6)
Kidney and urinary disorders	1 (0.6)	1 (0.6)
Hypotension	1 (0.6)	1 (0.6)
Sleep disorder	1 (0.6)	0
Bradycardia or tachycardia	1 (0.6)	0
Infections and infestations	1 (0.6)	0
Alanine aminotransferase increased	0	1 (0.6)
Nausea	0	1 (0.6)
Increased eosinophil count	0	1 (0.6)
Total protein decreased	0	1 (0.6)

## Data Availability

Anonymous participant data can be provided upon request to the corresponding author. Proposals will be reviewed and approved by the sponsor, security department, researcher, and staff on the basis of scientific merit and absence of competing interests. Once the proposal has been approved, data can be transferred through a secure online platform after the signing of a data access agreement and a confidentiality agreement.

## Supplementary Materials

The following supporting information can be downloaded at https://www.mdpi.com/article/10.3390/antibiotics13060476/s1. Table S1: Demographic and baseline characteristics of patients who received any dose of a study drug. Table S2: List of the trial sites and the principal investigators. Table S3: Evaluation criteria for the overall outcome at the TOC visit.
