# Supplementary material for: A New “Non-Traditional” Antibacterial Drug Fluorothiazinone—Clinical Research in Patients with Complicated Urinary Tract Infections
Source: Antibiotics (Basel). 2024 May 22;13(6):476. doi: 10.3390/antibiotics13060476 (PMC11200362; doi:10.3390/antibiotics13060476)
Supplement: Supplementary file 1 [file antibiotics-13-00476-s001.zip › antibiotics-2990148-supplementary.pdf]

Table S1: Demographic and baseline characteristics of patients who received any dose of study drug.

	FT/Cefepime<br>N = 180<br>n (%)	Placebo/Cefepime<br>N = 177<br>n (%)
Age, mean (SD), y	55.6 ± 18.6 (18.0-89.0)	58.6 ± 16.8 (20.0 – 86.0)
Age group, y		
<65	93 (51.5)	94 (52.8)
65 to <75	58 (32.4)	54 (30.6)
≥75	29 (16.1)	29 (16.6)
Sex		
Male	234 (45.3)	232 (44.8)
Female	282 (54.7)	286 (55.2)
Weight, mean (SD), kg	76.0 ± 15.0 (47.2-130.5)	76.7 ± 13.4 (45.3-130.0)
Height, mean (SD), cm	169.0 ± 9.9 (142.0-193.4)	168.2 ± 10.2 (144.0-196.1)
Body mass index, mean (SD)	26.7 ± 5.4 (16.6 – 45.2)	27.9 ± 5.5 (17.4 – 45.3)
eGFR at baseline, mean (SD), mL/min/1.73 m2	72.62 (22.0)	72.43 (23.8)
eGFR group at baseline, mL/min/1.73 m2		
Severe (<30)	1 (0.6)	1 (0.6)
Moderate (30-59)	35 (19.4)	37 (20.8)
Mild (60-89)	98 (54.4)	91 (51.8)
Normal (≥90)	46 (25.6)	47 (26.8)
Prior antibiotic therapy		
Short-acting antibiotic up to 24 h	12 (6.7)	9 (5.3)
No prior antibiotic therapy	168 (93.3)	168 (94.7)
Charlson Comorbidity Index (CCI) score at baselinee		
<3	113 (62.5)	109 (61.3)
≥3	68 (37.5)	68 (38.7)
Presence of concurrent bacteremia at baseline	0	1 (0.6)
Diabetes at baseline	23 (12.6)	25 (14.1)
Enterobacterales baseline pathogen, extended-spectrum β-lactamase producing	74 (41.2)	72 (40.6)

Table S2: List of the trial sites and the principal investigators.

Trial sites №	Name of the trial sites	Principal investigator
1	Private educational institution Clinical hospital» Russian Railways-Medicine of St. Petersburg	V. B. Shunkov
3	Eco-Safety Medical Center, St. Petersburg	D. S. Gorelov<br>D. S. Zlobin
5	State budgetary healthcare institution Vsevolozhsk Clinical Interdistrict Hospital, Vsevolozhsk	E. N. Matevosyan
7	Baltic Medicine Medical Center, St. Petersburg	M. O. Bushara
11	City Pokrovskaya Hospital of St. Petersburg	A. I. Gorelov
12	City Hospital №26 of St. Petersburg	A. A. Mangushlo
17	New clinic ABIA, St. Petersburg	O. F. Cagan

19	Hospital of the Holy Great Martyr George of St. Petersburg	A. N. Bulkin
21	City Hospital №15 of St. Petersburg	K. A. Dunec
22	Ryazan State Medical University named after Academician I.P. Pavlova, Ryazan	V. B. Filimonov
25	Hospital of Wars Veterans of Rostov Region	N. V. Budnik
27	Nizhny Novgorod Regional Clinical Hospital named after N.A. Semashko, Nizhny Novgorod	V. N. Krupin
29	Professor's Clinic, Perm	V. V. Vilesova
31	Department of Urology, Medical and Sanitary Unit 70 of Passazhiravtotrans, Saint Petersburg, St. Petersburg	V. A. Zubarev

Table S3: Evaluation criteria for the overall outcome at the TOC visit.

	Clinical response	Microbiological response
Overall success	Clinical cure	Microbiological eradication
		Presumed sustainable microbiological eradication
No effect	Recurrence/ Clinical inefficacy	Microbiological recurrence
		Colonization
		Microbiological eradication
		Presumed sustainable microbiological eradication
		Microbiologically uncertain response
	Clinical cure/ Clinically uncertain response	Microbiological recurrence
		Colonization
Uncertain outcome	There is insufficient data to determine cure or inefficacy to the patient	
